# Identifying a new microRNA signature as a prognostic biomarker in colon cancer

**DOI:** 10.1371/journal.pone.0228575

**Published:** 2020-02-12

**Authors:** Yunxia Lv, Jinzhong Duanmu, Xiaorui Fu, Taiyuan Li, Qunguang Jiang

**Affiliations:** 1 Department of Thyroid Surgery, The Second Affiliated Hospital of Nanchang University, Nanchang, Jiangxi, People's Republic of China; 2 Department of Gastrointestinal Surgery, The First Affiliated Hospital of Nanchang University, Nanchang, Jiangxi, People's Republic of China; 3 Queen Mary School, Medical Department, Nanchang University, Nanchang, Jiangxi, People's Republic of China; University of Alabama at Birmingham, UNITED STATES

## Abstract

**Background:**

The aim was to identify a novel prognostic miRNA signature for colon cancer (CC) *in silico*.

**Methods:**

Data on the expression of miRNAs and relevant clinical information for 407 patients were obtained from The Cancer Genome Atlas (TCGA), and the samples were randomly split into a validation set (n = 203) and training set (n = 204). The differential expression of miRNAs between normal tissues and patients with CC was analyzed. We detected a miRNA expression signature in the training dataset by using a Cox proportional hazard regression model. Then, we verified the signature in the validation set. Association of the miRNA signature with overall survival was assessed in the validation cohort and combined cohort by log-rank test and based on Kaplan-Meier curves. The receiver operating characteristic and disease-free survival analyses were performed to evaluate the miRNA signature of CC in the combined cohort. Multivariate and univariate Cox analyses related to survival for the miRNA signature were performed, and a nomogram was built as a prognostic model for CC. To explore the function of target genes of the miRNA signature, Gene Ontology analysis and Kyoto Encyclopedia of Genes and Genomes pathway analysis were used.

**Results:**

Between the matched normal tissues and colon cancer tissues, 267 differentially expressed miRNAs were detected, and a single-factor CoxPH model showed that 13 miRNAs were related to overall survival in the training cohort. Then, a five-miRNA signature was identified using a CoxPH regression model with multiple factors. The five-miRNA signature had significant prognostic value in the training cohort and was validated in the validation cohort and combined cohort. A total of 193 target genes of the miRNA signature were identified. According to the results of functional analysis of the target genes, the signaling pathways MAPK, AMPK and PI3K-Akt, focal adhesion, and microRNAs in cancer were remarkably enriched.

**Conclusion:**

A five-miRNA signature had increased prognostic value for CC, which may provide important biological insights for the discovery and development of molecular predictors to improve the prognosis of patients with CC.

## Introduction

Colon cancer (CC) is a common malignant tumor of the digestive tract worldwide. The incidence ranks third among all malignant tumors, and over 1 million new cases occur per year.[1] Despite continuous improvements in surgery, chemotherapy and radiotherapy, approximately 50% of patients still have recurrence and metastasis within 5 years, which leads to death.[2] Although great progress has been made in understanding the pathogenesis and molecular mechanisms of CC, there is no effective molecular diagnostic approach to predict prognosis. Therefore, it is essential to identify a prognostic detection molecular model for treatment planning, outcome prediction and patient evaluation.

As important epigenetic regulatory factors, microRNAs (miRNAs), which are endogenous noncoding single-stranded RNA molecules composed of 18 to 24 nucleotides, are closely related to tumor development. The function of miRNAs, by binding to target mRNAs, is to regulate posttranscriptional gene expression to inhibit the translation process or induce mRNA degradation.[3] Based on these functions, miRNAs are involved in different cellular biological events (e.g., cell apoptosis, growth, differentiation and proliferation,[4] and tumorigenesis and progression[5]). Growing evidence demonstrates that dysregulated miRNAs are vital to colon cancer pathogenesis and progression. Several miRNAs (e.g., miR-224,[6] miR-146a,[7] miR-21,[8] miR-106a[9] and miR-301a[10]) can facilitate the invasion and migration of cancer cells and maintain resistance to chemotherapy and the characteristics of cancer stem cells. Additionally, other miRNAs (e.g., miR-15a,[11] miR-21,[12] and miR-29a[13]) may have prognostic value in colon cancer. Several miRNAs have been identified to predict prognosis in colon cancer, but there have been inconsistencies in previous studies. MiRNA expression signatures associated with prognosis have been identified in malignancy.[14] Thus, in our study, a novel miRNA signature capable of predicting prognosis in CC patients, was identified and validated in TCGA (The Cancer Genome Atlas) database.

## Materials and methods

### Data processing

Clinical information on the colon cancer cohort and miRNA sequencing data (level 3, reads per kilobase million; RPKM) were collected from the TCGA database (https://cancergenome.nih.gov/). Inclusion criteria are presented below: (1) pathological type was adenocarcinoma; (2) miRNA sequencing data and clinical data were collected; (3) samples with prognostic information; and (4) follow-up days no less than 30. A total of 415 samples were included in this study, covering 8 matched normal tissues and 407 colon cancer tissues. A total of 407 colon cancer cases were randomly split into a validation cohort (203 cases) and a training cohort (204 cases) by a R package called “caret”. Since the data were obtained from the TCGA database, no further approval was required from the Ethics Committee.

The limma package in R was used to analyze the differences in expression levels between CC tissues and matched normal tissues. The Wilcoxon test was used to identify miRNAs with significant expression differences between CC tissues and matched normal tissues. Differentially expressed miRNAs with log_2_|FC|>1.0 and FDR adjusted P<0.05 were significant.

### Identification and survival analysis of a miRNA signature in the training cohort

To determine the correlation of survival time with miRNAs differentially expressed in CC, a single-factor Cox proportional hazard (CoxPH) regression model was fitted to the training cohort data. The statistical significance was p<0.05. After adjustment, a CoxPH regression model with multiple factors was employed to identify the CC survival assessment model miRNA signature. Model coefficients were used to calculate risk scores (RS), and log-rank test was used in the training cohort to test patients with high and low risk. The survminer package in R was used to calculate the cutoff value for the RS. The correlation between overall survival and disease-free survival with miRNA signature expression was plotted using the Kaplan-Meier method and log-rank test.

### Validation of miRNA signature in the validation cohort and combined cohort

Similarly, with the coefficients used in the model for the validation cohort and combined cohort, we calculated a risk score, and subsequently drew a comparison between high- and low-risk patients by Kaplan-Meier analysis and log-rank test. In addition, receiver operating characteristic (ROC) and disease-free survival analyses were performed to evaluate the prognostic potential of the miRNA signature for CC in the combined cohort. Multivariate and univariate Cox analyses of the miRNA signature related to survival were performed, and a nomogram was built as a prognostic model for CC.

### Target gene prediction of the prognostic miRNA signature

Target genes of the prognostic miRNAs were predicted with the use of online analysis tools, miRTarBase (http://mirtarbase.mbc.nctu.edu.tw/php/index.php), miRDB (http://www.mirdb.org/miRDB/), and TargetScan (http://www.targetscan.org/). To improve the reliability of the bioinformatics analysis, the overlapping target genes were identified by Venn diagram. Subsequently, using the Database for Annotation, Visualization and Integrated Discovery (DAVID) bioinformatics tool (https://david.ncifcrf.gov/), we further studied overlapping genes. DAVID is an online bioinformatics resource designed to provide users with a comprehensive set of functional annotation tools to understand the biological mechanisms associated with large lists of genes/proteins. For the target genes, Kyoto Encyclopedia of Genes and Gene Ontology (KEGG) pathway enrichment analyses and Gene Ontology (GO) analysis were performed. The cutoff criteria included a gene count ≥5 and a P-value < 0.05.

### Statistical analysis

Data are represented as mean±standard deviation (SD). Univariate Cox proportional hazard regression model and multivariate Cox proportional hazard regression model were used to evaluate the prognostic significance of miRNA signals. All statistical analyses were conducted by SPSS 22.0 software (SPSS Inc., Chicago, IL, USA). P <0.05 was statistically significant.

## Results

### Identification of differentially expressed miRNAs in colon cancer

**Fig 1:**
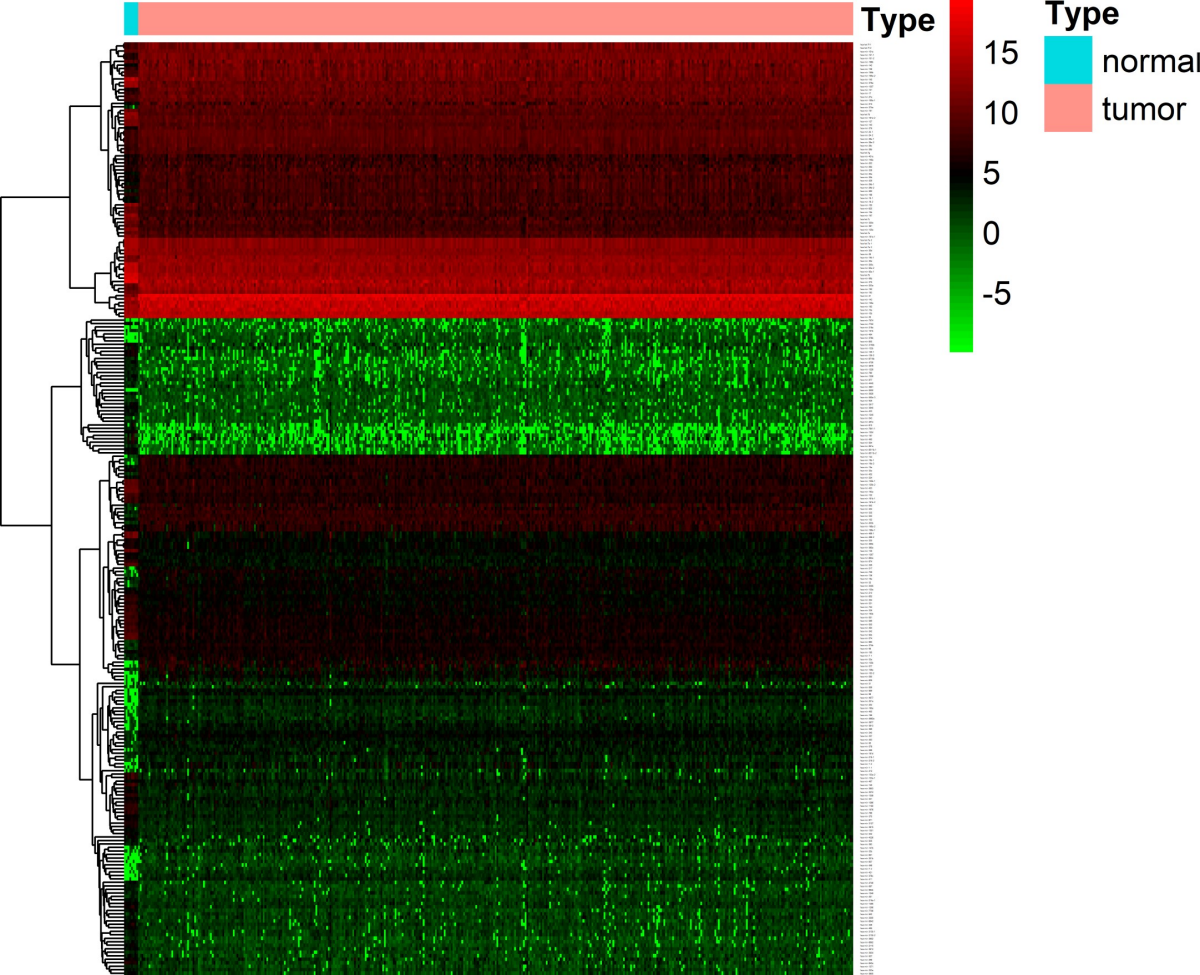
Heatmap of miRNA expression in CC. The horizontal axis above indicates the groups of samples. The vertical axis on the left shows the cluster of different miRNAs, and the right axis shows the miRNA name. Red indicates high expression, and green indicates low expression. miRNA, microRNA; CC, colon cancer.

All 407 colon cancer cases were randomly split into a validation cohort (203 cases) and a training cohort (204 cases). There were no significant differences in follow-up results, follow-up time, vascular invasion, family history, tumor stage, race or gender between the two groups. The clinical characteristics of the two groups are listed in Table 1. Following predetermined cutoff criteria (FDR adjusted P < 0.05 and |log 2 FC| > 1.0), a total of 267 differentially expressed miRNAs were detected between the matched normal tissues and colon cancer tissues including 141 downregulated and 126 upregulated miRNAs (S1 Table). The result is presented as a heatmap to verify whether the P value and |log 2 FC| are consistent in different tests (Fig 1).

**Table 1:** The main clinical and pathological characteristics of the 407 CC patients.

		combined cohort (407)	training (204)	validation (203)	P
age	≧60	287	145	142	0.444
	<60	120	59	61	
gender	male	217	110	107	0.442
	female	190	94	96	
T	T1+T2	82	41	41	0.539
	T3+T4	325	163	162	
N	N0	236	122	114	0.26
	N1+N2	171	82	89	
M	M0	298	152	145	0.49
	M1	60	30	30	
	MX	45	19	26	
	unknown	5	3	2	
stage	Stage I/II	220	113	107	0.307
	Stage III/IV	176	85	91	
	unknow	11	6	5	
venous invasion	no	225	114	111	0.513
	yes	140	72	68	
	NA	42	18	24	
lymphatic invasion	no	263	133	130	0.487
	yes	88	45	43	
	NA	56	26	30	
New event	no	319	164	155	0.192
	yes	88	40	48	
New event time (days)		791.17±712.61	806.11±674.50	773.75±748.61	0.379
survival status	alive	319	160	159	
	dead	88	44	44	0.538
survival time (days)		887.73±751.11	886.15±695.39	889.32±803.21	0.133

Clinicopathological parameters were compared between training set and validation set by the χ2 test and the unpaired two-tailed Student’s t test. The P value refers to the χ^2^ test except for new event time and survival time. The P value of new event time and survival time refers to the unpaired two-tailed Student’s t test. CC, colon cancer; T, tumor; N, node; M, metastasis.

### Generation and validation of a miRNA signature

**Fig 2:**
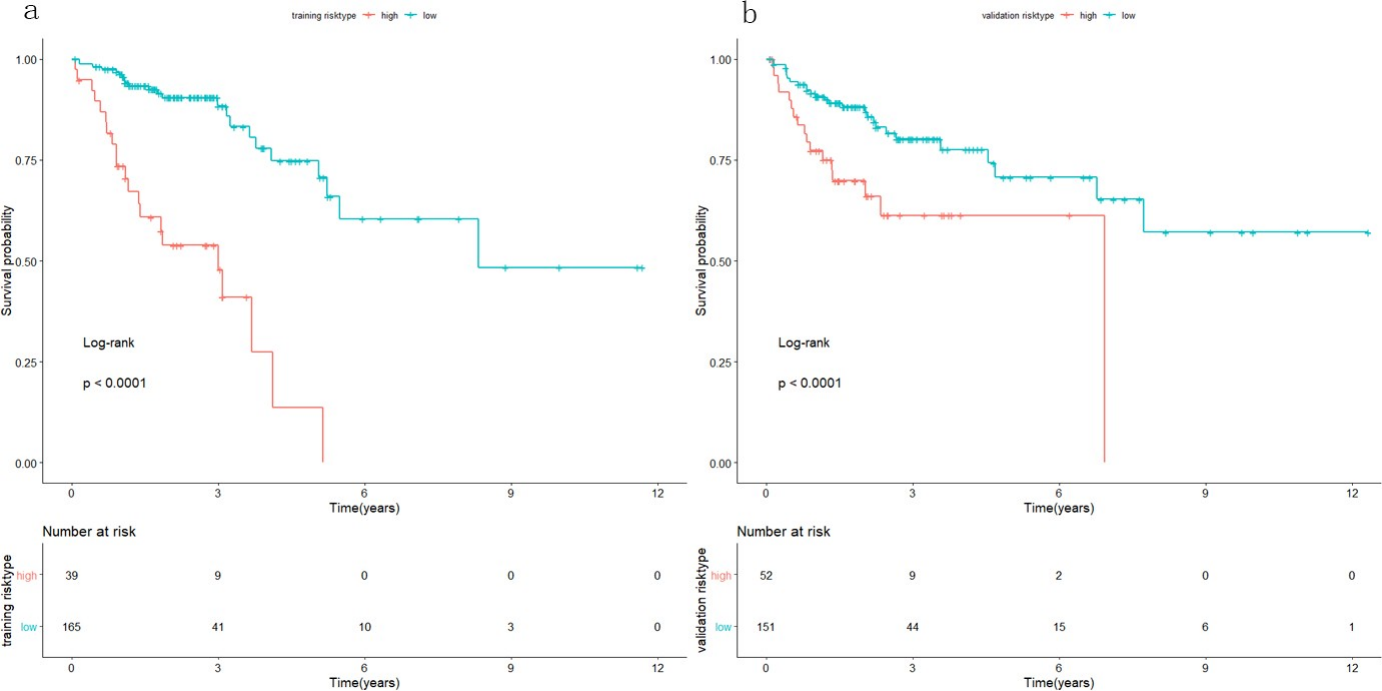
Survival analysis in the training and validation cohorts. In the training (a) and validation (b) cohorts, the patients were split into groups of high and low risk. Survival analysis was conducted by Kaplan‐Meier analysis and log-rank test.

**Fig 3:**
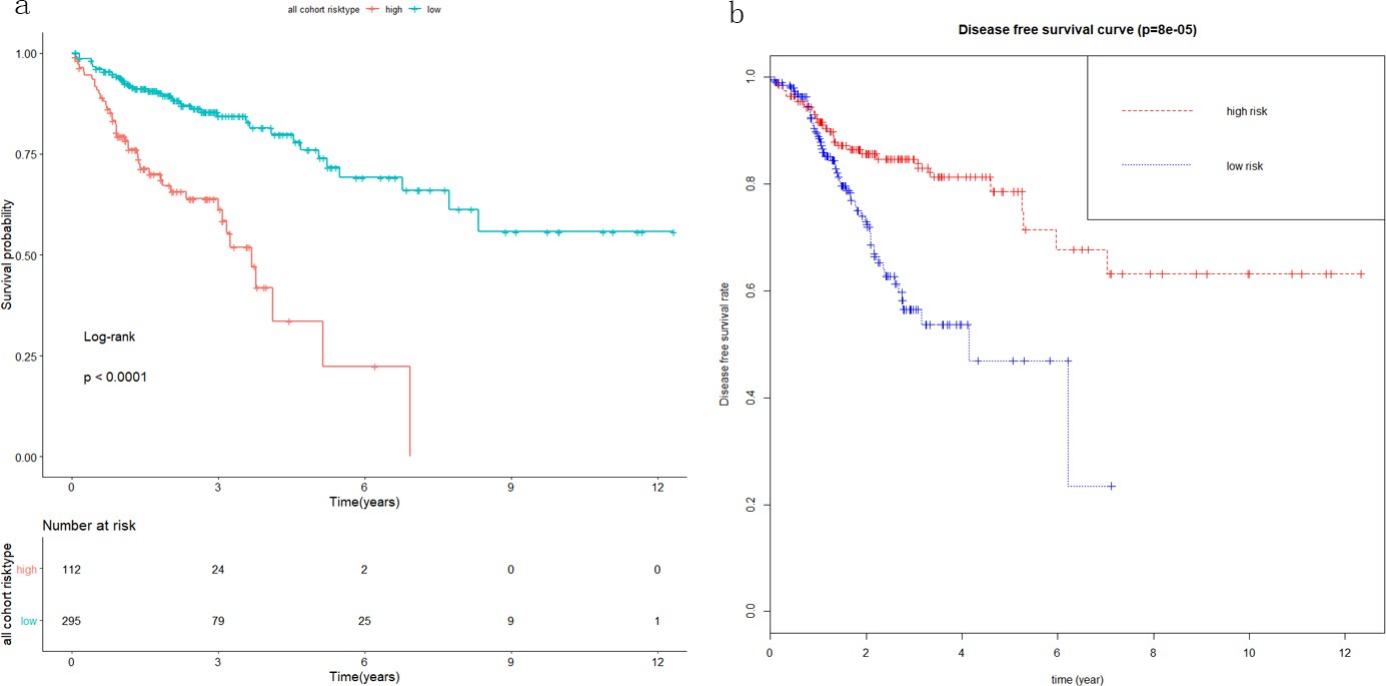
Survival and disease-free survival analyses in the combined cohort. The survival curve (a) and disease-free survival (b) were calculated for the combined cohort according to the miRNA signature by Kaplan-Meier and log-rank.

**Fig 4:**
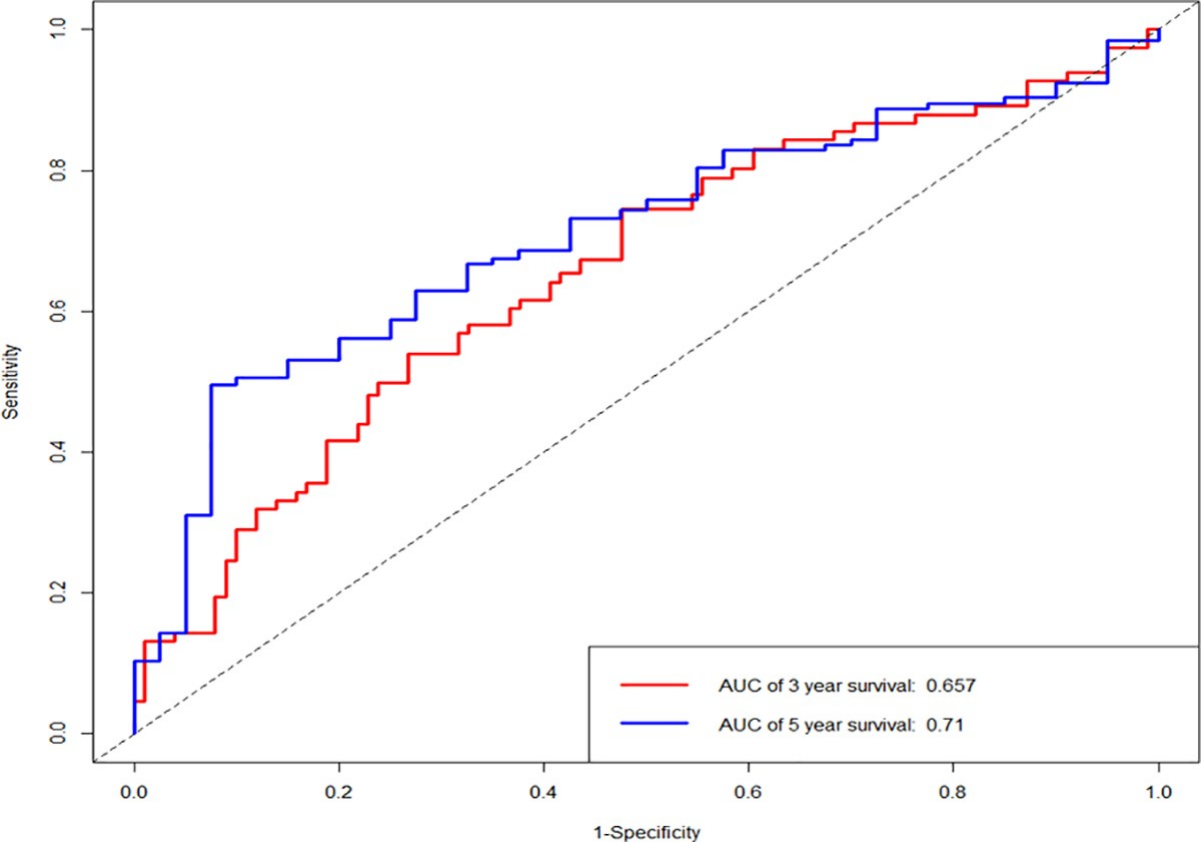
ROC curve. The ROC curve of the miRNA signature for 3-year and 5-year survival probability in the combined cohort. ROC, receiver operating characteristic; miRNA, microRNA.

**Fig 5:**
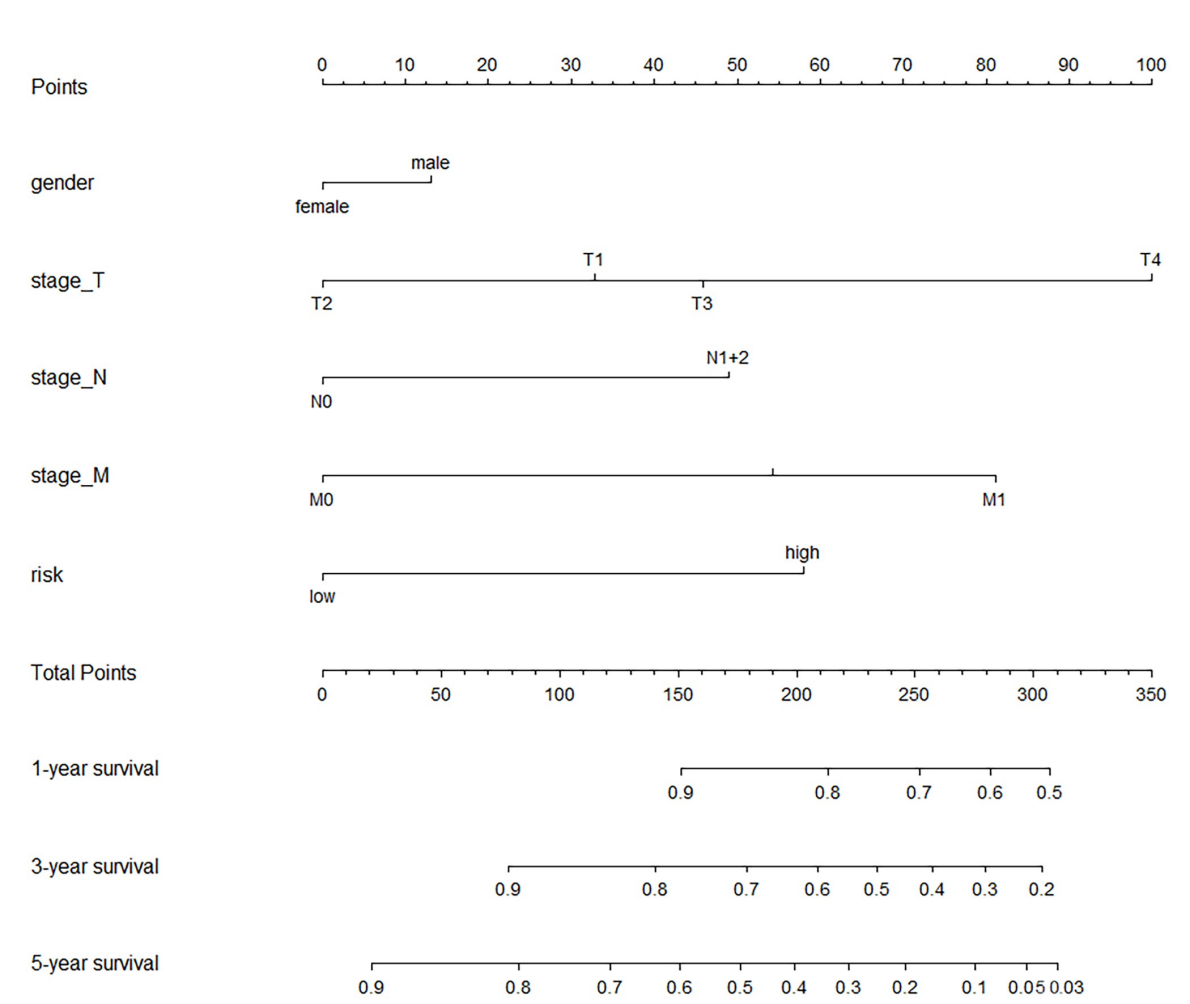
The nomogram. A nomogram for predicting the survival probability of CC patients with 1-, 3- and 5-year OS. CC, colon cancer; OS, overall survival.

A single-factor CoxPH regression model fitted to the training cohort produced 13 miRNAs (Table 2). A set of miRNA markers associated with survival was identified after the CoxPH regression model with a multiple factor analysis. Combining the miRNA signature with its coefficients in the penalty function, the following equation was obtained: RiskScore = 0.2801*exp(hsa−mir−891a)+0.1982*exp(hsa−mir−187)+(−0.4228)*exp(hsa−mir−3677)+0.3225*exp(hsa−mir−615)+0.6009*exp(hsa−mir−101−2). The risk score for each patient in the training cohort was calculated, and the patients were divided into a low-risk group and a high-risk group by using the optimal cutoff value of the survminer package in R. There was a significant difference between the high-risk group and the low-risk group (P < 0.001), as shown in Fig 2A. Similar significant differences were observed between the high-risk and low-risk groups when the same miRNA signature equation was applied to the validation cohort (P < 0.001), as demonstrated in Fig 2B. The survival curve and disease-free survival were calculated in the combined cohort according to the miRNA signature by Kaplan-Meier analysis and log-rank test, and both were significantly different between the high-risk group and the low-risk group (P < 0.001) (Fig 3A and 3B). In addition, we conducted 3-year and 5-year survival analyses of the miRNA signature using ROC and calculated AUC to evaluate the identification ability of the miRNA signature (Fig 4). The three-year survival AUC of the miRNA signature was 0.657. The five-year survival AUC of the miRNA signature was 0.71. Our results suggest that miRNA signaling may be a prognostic model for predicting CC survival. The following clinical characteristics were considered: age, sex, T staging, lymph node status, metastasis, staging, lymphatic infiltration and venous infiltration. Single-factor Cox regression and multifactor Cox regression analyses were used to detect the influence of the 5-miRNA signature on OS. In univariate analysis, T stage, lymph node status, metastasis, stage, lymphatic infiltration and venous infiltration in colon cancer patients were associated with OS. Multivariate analysis showed that the 5-miRNA signature (HR = 1.9, P = 0.03) was an independent prognostic factor for colon cancer patients (Table 3). Additionally, a nomogram combining the risk score with clinicopathological parameters was built to predict the prognosis of colon cancer patients (Fig 5).

**Table 2:** Thirteen miRNAs were filtered by the single-factor CoxPH regression model.

Gene	HR	z	HCI	LCI	P value
hsa-mir-891a	1.43770557	3.75114291	1.73800957	1.18928995	0.00017603
hsa-mir-187	1.46471517	3.62590004	1.80032254	1.1916701	0.00028796
hsa-mir-3677	0.59141546	-3.5243229	0.79204318	0.44160755	0.00042457
hsa-mir-615	1.29354218	2.43078308	1.59188018	1.05111641	0.01506623
hsa-let-7c	1.37425018	2.4159671	1.77857545	1.06184056	0.01569348
hsa-mir-552	0.8524886	-2.3214254	0.97545615	0.74502254	0.0202639
hsa-mir-101-1	1.89297266	2.3124699	3.25118467	1.10216609	0.0207518
hsa-mir-101-2	1.89471139	2.30488128	3.26251712	1.1003563	0.02117322
hsa-mir-125b-2	1.36793527	2.18546453	1.8117221	1.03285537	0.0288548
hsa-mir-664a	1.59675242	2.18076427	2.43163036	1.04852215	0.02920086
hsa-mir-125b-1	1.36451708	2.16450621	1.80801764	1.02980569	0.03042552
hsa-mir-218-1	1.42318528	2.11057397	1.97508681	1.02550243	0.03480895

CoxPH, Cox proportional hazards; HR, hazard ratio.

**Table 3:** A multivariate analysis of survival with clinical factors and the miRNA signature.

	univariate Cox		multivariate Cox	
	HR(95% CI)	P value	HR(95% CI)	P value
T (T3+T4 vs T1+T2)	3.56(1.45–8.90)	<0.01*		
N (N1+2 vs N0)	3.13(2.01–4.86)	<0.01*		
M (M1 vs M0)	5.23(3.26–8.38)	<0.01*	2.07(1.1–3.89)	0.02*
stage (stage III+IV vs stage I+II)	3.76(2.34–6.05)	<0.01*	5.53(1.5–20.43)	0.01*
age (≥60 vs <60)	1.02(0.99–1.03)	0.08		
venous invasion (yes vs no)	2.76(1.75–4.37)	<0.01*		
lymphatic invasion (yes vs no)	2.38(1.51–3.75)	<0.01*		
gender (female vs male)	1.23(0.81–1.89)	0.33		
risk (high vs low)	2.85(1.81–4.51)	<0.01*	1.9(1.08–3.36)	0.03*

* P<0.05. CI, confidence interval; HR, hazard ratio; miRNA, microRNA.

#### Target prediction and functional analysis

**Fig 6:**
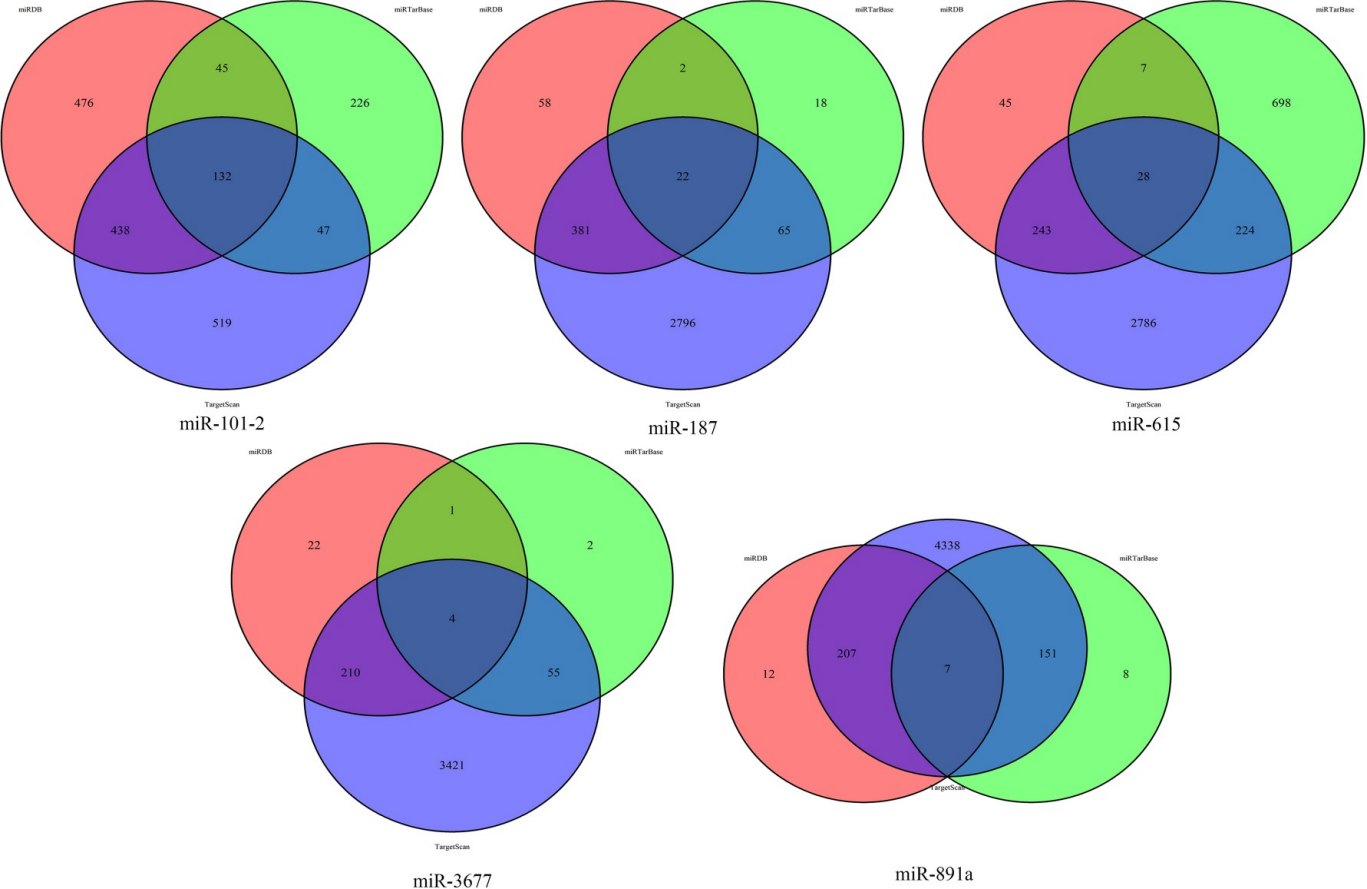
The overlapping target genes. The overlapping target genes were predicted using the TargetScan, miRDB, and miRTarBase online analysis tools.

**Fig 7:**
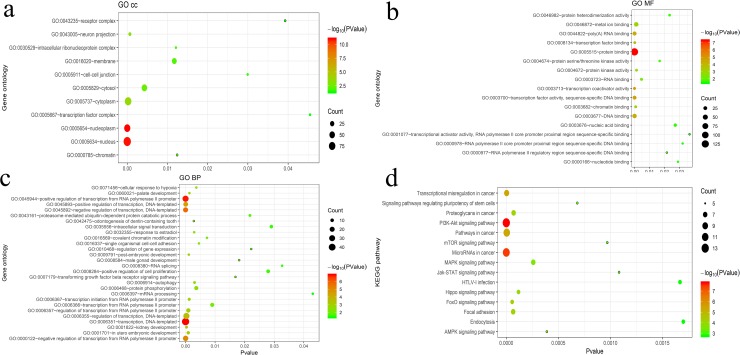
Biological function and KEGG pathway analyses of target genes. (a) The enriched GO cellular component terms of target genes. (b) The enriched GO molecular function terms of target genes. (c) The enriched GO biological process terms. (d) The enriched pathways of the target gene determined by KEGG pathway analysis. GO, Gene ontology; KEGG, Kyoto Encyclopedia of Genes and Genomes.

The target genes of five miRNAs (miR-3677, miR-615, miR-101-2, miR-187 and miR-891a) were predicted with the use of the TargetScan, miRDB, and miRTarBase online analysis tools. A total of 132 overlapping genes for miR-101-2, 22 overlapping genes for miR-187, 28 overlapping genes for miR-615, 7 overlapping genes for miR-891a, and 4 overlapping genes for miR-3677 were detected (Fig 6). Subsequently, enrichment analysis was carried out to elucidate the biological functions of the consensus target genes. KEGG pathway analysis showed that the MAPK signaling pathway, proteoglycan in cancer, focal adhesion, transcription disorders in cancer, pathways in cancer, microRNAs in cancer, and the PI3k-akt signaling pathway were significantly enriched. In addition, the enriched GO terms were related to apoptotic processes, cell proliferation and autophagy (Fig 7).

## Discussion

Colon cancer is a common digestive tract malignant tumor with high morbidity and mortality. Although great progress has been made in understanding the pathogenesis and molecular mechanisms of CC, there is no effective molecular diagnostic approach to predict poor prognosis. For their stability and widespread distribution in human tissues, miRNAs are considered a new class of disease biomarkers.[15] Mounting evidence has demonstrated that miRNAs seem to be therapeutic targets and prognostic indicators in CC.[16] Some miRNAs have been shown to be critical to CC progression and tumorigenesis by regulating biological processes. Several studies have detected miRNAs with prognostic implications (e.g., miR-10b,[17] miR-17-92a cluster,[18] miR-29a,[13] miR-31,[19] miR-21,[20] miR-182,[21] and miR-185[22]). Here, a five-miRNA signature was detected as an individual prognostic factor for colon cancer based on the TCGA database, a source of “big data” with sufficient sample size and clinical information.

Additionally, the target genes of these five miRNAs were predicted, and with the use of bioinformatics methods, the enriched KEGG pathways and Gene Ontology terms of the target genes were analyzed. A recent study retrieved TCGA data and reported that a seven-miRNA signature (mir-652, mir-505, mir-328, mir-197, mir-181a-1, mir-32 and let-7a-2) was related to the prognosis of CC as demonstrated by the Kaplan-Meier method and the principal component model.[23] In addition, another study unveiled a novel 16-miRNA signature able to prognosticate stage II and III colon cancer outcome using lasso regression.[24] However, these two studies either had a small sample size or lacked validation sets. Here, by using the Wilcox test and the limma package, the TCGA-COAD cohort was analyzed to identify differentially expressed miRNAs. Then, the TCGA-COAD cohort was randomly split into a validation set and training set. Subsequently, the five-miRNA signature was identified in the training set using single factor and multifactor CoxPH regression. Moreover, the five-miRNA signature was validated in the validation set and the combined cohort. Multifactor Cox analysis suggested that the five-miRNA signature was an individual colon cancer prognostic factor.

Three were three downregulated (miR-891a, miR-615, miR-187) and two upregulated (miR-3677, miR-101-2) miRNAs in the five-miRNA signature. miR-187 expression in different human tumors is inconsistent, and its high expression promotes metastasis and invasion in breast cancer cells and is an independent risk factor for prognosis.[25] In ovarian cancer, its high expression can promote tumor progression in the early stage and inhibit epithelial-mesenchymal transition by acting on disability homolog 2 (DAB2) during the later stages.[26] This may indicate that miR-187 plays a dual role in tumorigenesis and progression. It has been reported that miR-187 suppresses Smad-mediated epithelial-mesenchymal transition in colorectal cancer.[27] Additionally, miR-187 can inhibit tumor growth and invasion by directly targeting CD276 in colorectal cancer.[28] Gao et al reported that miR-615 targeted AKT2 and inhibited AKT2-mediated cell proliferation in pancreatic ductal adenocarcinoma.[29] Additionally, miR-615 inhibited cell proliferation, invasion, and metastasis by targeting IGF2 in hepatocellular cancer.[30] As a member of the miR-888 cluster, miR-891a, located on human chromosome Xq27.3, can promote prostate cancer cell growth and invasion by directly targeting TIMP2.[31] Combined with several other miRNAs, miR-3677 can predict the prognosis of hepatocellular carcinoma.[32] The results of these studies are consistent with our data mining. miR-101-2 appears to be a tumor suppressor gene by suppressing growth, proliferation and migration and inducing apoptosis in a host of cancer,[33] including colon cancer.[34] miR-101-2 was upregulated, and it seems to be an oncogene in CC. Thus, the conflicting function of miR-101-2 requires subsequent investigations.

Gene Ontology and KEGG pathway analyses of the target genes were performed, and several key signaling pathways (mTOR, Hippo, MAPK, AMPK, and PI3K-Akt signaling pathways, as well as miRNAs in cancer and focal adhesion) were regulated. These signaling pathways are related to tumorigenesis and progression in CC. For instance, PI3K/Akt/mTOR can promote cell invasion and migration in CC and has potential to be a therapeutic target.[35] Thus, it is necessary to investigate these predictions, which are likely to offer novel therapeutic targets in CC.

This study still has limitations. First, the of five-miRNA signature should be verified in other external independent cohorts. Second, there is a lack of validation in clinical practice.

In summary, this study successfully detected and validated a 5-miRNA signature in patients with CC in TCGA. These findings require further clinical validation. The molecular mechanisms of the five miRNAs should be explored in CC development.

## Supporting information

S1 Table Differentially expressed miRNA. 267 miRNAs expressed differentially were detected between matched normal tissues and colon cancer tissues, covering 141 declined and 126 risen miRNAs. miRNA, microRNA.
(DOCX)
