# Supplementary material for: Identifying a new microRNA signature as a prognostic biomarker in colon cancer
Source: PLoS One. 2020 Feb 12;15(2):e0228575. doi: 10.1371/journal.pone.0228575 (PMC7015317; doi:10.1371/journal.pone.0228575)
Supplement: S1 Table — 267 miRNAs expressed differentially were detected between matched normal tissues and colon cancer tissues, covering 141 declined and 126 risen miRNAs. miRNA, microRNA. (DOCX) [file pone.0228575.s001.docx]

| Gene | ConMean | TreatMean | logFC | p Value | FDR |
| --- | --- | --- | --- | --- | --- |
| hsa-mir-504 | 43.7106368 | 0.5651018 | -6.2733298 | 6.74E-07 | 5.94E-06 |
| hsa-mir-486-2 | 2010.88615 | 41.675624 | -5.5924838 | 1.35E-06 | 5.94E-06 |
| hsa-mir-486-1 | 1955.82441 | 41.849391 | -5.5464264 | 1.35E-06 | 5.94E-06 |
| hsa-mir-328 | 687.072413 | 16.3510697 | -5.3930052 | 1.27E-06 | 5.94E-06 |
| hsa-mir-139 | 1160.39344 | 29.1239651 | -5.3162634 | 1.29E-06 | 5.94E-06 |
| hsa-mir-6511b-2 | 28.0218475 | 0.70401272 | -5.3148068 | 1.04E-06 | 5.94E-06 |
| hsa-mir-6511b-1 | 25.42381 | 0.70285526 | -5.1768088 | 9.68E-07 | 5.94E-06 |
| hsa-mir-129-1 | 56.8023663 | 1.85151679 | -4.9391715 | 1.32E-06 | 5.94E-06 |
| hsa-mir-197 | 7853.80713 | 267.76686 | -4.8743428 | 1.27E-06 | 5.94E-06 |
| hsa-mir-129-2 | 57.9123875 | 2.04396608 | -4.8244288 | 1.44E-06 | 5.94E-06 |
| hsa-mir-187 | 38.3124108 | 1.53057121 | -4.6456697 | 0.00014436 | 0.00022209 |
| hsa-mir-490 | 89.1784069 | 3.65957884 | -4.6069449 | 3.89E-05 | 6.90E-05 |
| hsa-mir-1224 | 41.1749425 | 1.8746885 | -4.4570438 | 5.61E-07 | 5.94E-06 |
| hsa-mir-7702 | 23.5033993 | 1.08005614 | -4.4436912 | 0.00014411 | 0.00022209 |
| hsa-mir-766 | 139.644173 | 6.43268991 | -4.4401894 | 1.31E-06 | 5.94E-06 |
| hsa-mir-642a | 90.296115 | 4.23851701 | -4.4130324 | 1.27E-06 | 5.94E-06 |
| hsa-mir-1976 | 160.883079 | 8.12052644 | -4.3082955 | 1.29E-06 | 5.94E-06 |
| hsa-mir-133a-2 | 207.77176 | 10.7877447 | -4.2675344 | 1.69E-06 | 5.94E-06 |
| hsa-mir-1306 | 135.073193 | 7.30079955 | -4.2095431 | 1.27E-06 | 5.94E-06 |
| hsa-mir-149 | 249.4123 | 14.0415117 | -4.1507624 | 1.59E-06 | 5.94E-06 |
| hsa-mir-133a-1 | 209.201518 | 12.3307331 | -4.0845628 | 1.79E-06 | 5.94E-06 |
| hsa-mir-125a | 4761.09413 | 310.146312 | -3.9402723 | 1.27E-06 | 5.94E-06 |
| hsa-mir-145 | 39644.2 | 2673.89383 | -3.8900957 | 1.66E-06 | 5.94E-06 |
| hsa-mir-1180 | 159.445474 | 10.8015524 | -3.8837526 | 1.29E-06 | 5.94E-06 |
| hsa-mir-150 | 6682.9275 | 465.364595 | -3.8440469 | 1.35E-06 | 5.94E-06 |
| hsa-mir-1226 | 17.1861383 | 1.21114482 | -3.8268021 | 1.46E-06 | 5.94E-06 |
| hsa-mir-615 | 25.8630485 | 1.91746289 | -3.7536218 | 0.00616856 | 0.00778755 |
| hsa-mir-574 | 765.965575 | 59.2673292 | -3.6919706 | 1.35E-06 | 5.94E-06 |
| hsa-mir-4728 | 17.6385406 | 1.37609146 | -3.6800829 | 1.69E-06 | 5.94E-06 |
| hsa-mir-326 | 56.291964 | 4.41735891 | -3.671673 | 4.69E-05 | 8.17E-05 |
| hsa-mir-1296 | 64.4717638 | 5.17034332 | -3.6403355 | 1.61E-06 | 5.94E-06 |
| hsa-let-7d | 7622.517 | 612.918492 | -3.6365003 | 1.27E-06 | 5.94E-06 |
| hsa-mir-7641-1 | 10.6230725 | 0.85560979 | -3.6341043 | 1.50E-06 | 5.94E-06 |
| hsa-mir-3940 | 23.3169913 | 1.95695895 | -3.5746962 | 1.30E-06 | 5.94E-06 |
| hsa-mir-3615 | 58.546765 | 4.9402916 | -3.5669214 | 1.37E-06 | 5.94E-06 |
| hsa-mir-433 | 16.4255748 | 1.41056001 | -3.5416039 | 1.69E-06 | 5.94E-06 |
| hsa-mir-3917 | 20.3068139 | 1.74649631 | -3.5394284 | 2.35E-06 | 6.60E-06 |
| hsa-let-7b | 106443.091 | 9565.06271 | -3.4761641 | 1.27E-06 | 5.94E-06 |
| hsa-mir-3127 | 77.2651138 | 7.18287058 | -3.4271847 | 1.27E-06 | 5.94E-06 |
| hsa-mir-2110 | 26.7175088 | 2.50005288 | -3.417755 | 1.33E-06 | 5.94E-06 |
| hsa-mir-3150b | 37.04547 | 3.6523808 | -3.342388 | 3.07E-06 | 7.95E-06 |
| hsa-mir-193a | 1444.33275 | 143.979116 | -3.3264717 | 1.31E-06 | 5.94E-06 |
| hsa-mir-485 | 31.4228913 | 3.25531923 | -3.270945 | 1.69E-06 | 5.94E-06 |
| hsa-mir-4676 | 8.33755075 | 0.87373474 | -3.2543564 | 1.24E-06 | 5.94E-06 |
| hsa-mir-181a-1 | 4474.86763 | 473.258939 | -3.2411433 | 1.33E-06 | 5.94E-06 |
| hsa-mir-92b | 635.793463 | 67.6992353 | -3.2313467 | 1.41E-06 | 5.94E-06 |
| hsa-mir-1249 | 14.1472901 | 1.52622289 | -3.2124882 | 1.65E-06 | 5.94E-06 |
| hsa-mir-1228 | 8.10502213 | 0.88495451 | -3.1951409 | 1.80E-06 | 5.94E-06 |
| hsa-mir-7706 | 20.7464325 | 2.32536588 | -3.1573336 | 1.27E-06 | 5.94E-06 |
| hsa-mir-375 | 249729.188 | 28072.3706 | -3.1531416 | 1.29E-06 | 5.94E-06 |
| hsa-mir-193b | 303.707438 | 35.7659443 | -3.0860238 | 1.41E-06 | 5.94E-06 |
| hsa-mir-99b | 122572.748 | 14526.5627 | -3.076873 | 1.35E-06 | 5.94E-06 |
| hsa-mir-423 | 1152.12145 | 137.684488 | -3.0648549 | 1.29E-06 | 5.94E-06 |
| hsa-mir-3928 | 12.5625809 | 1.58740243 | -2.9843931 | 3.39E-06 | 8.53E-06 |
| hsa-mir-6892 | 14.480025 | 1.9248235 | -2.911266 | 1.73E-06 | 5.94E-06 |
| hsa-mir-378a | 10467.2911 | 1414.10441 | -2.8879276 | 1.27E-06 | 5.94E-06 |
| hsa-mir-3605 | 22.5539144 | 3.14359725 | -2.8428895 | 2.30E-06 | 6.57E-06 |
| hsa-mir-125b-1 | 913.20385 | 130.440988 | -2.8075397 | 1.82E-06 | 5.94E-06 |
| hsa-mir-125b-2 | 922.447613 | 133.698573 | -2.7864829 | 1.87E-06 | 5.97E-06 |
| hsa-mir-760 | 8.252691 | 1.21461238 | -2.7643686 | 4.71E-06 | 1.10E-05 |
| hsa-mir-671 | 48.1046725 | 7.10590655 | -2.7590864 | 1.39E-06 | 5.94E-06 |
| hsa-let-7c | 3159.901 | 470.698498 | -2.7470042 | 1.71E-06 | 5.94E-06 |
| hsa-mir-133b | 29.4275148 | 4.48759456 | -2.7131515 | 0.00030767 | 0.00044624 |
| hsa-mir-484 | 346.3315 | 53.3233748 | -2.6993136 | 1.50E-06 | 5.94E-06 |
| hsa-mir-874 | 132.093275 | 20.3516393 | -2.6983401 | 1.29E-06 | 5.94E-06 |
| hsa-mir-6842 | 18.3569044 | 2.84391322 | -2.6903734 | 2.01E-06 | 6.12E-06 |
| hsa-mir-1307 | 11030.7025 | 1730.27771 | -2.6724492 | 1.35E-06 | 5.94E-06 |
| hsa-mir-6715b | 8.07071613 | 1.26992944 | -2.6679483 | 7.94E-06 | 1.71E-05 |
| hsa-mir-1468 | 29.918885 | 4.86046447 | -2.6218903 | 1.90E-06 | 5.97E-06 |
| hsa-mir-487a | 8.4961395 | 1.41311339 | -2.5879302 | 2.58E-06 | 7.12E-06 |
| hsa-mir-296 | 38.2054338 | 6.45347569 | -2.5656296 | 7.74E-06 | 1.70E-05 |
| hsa-mir-324 | 197.11875 | 33.3141988 | -2.5648559 | 1.37E-06 | 5.94E-06 |
| hsa-mir-432 | 34.162765 | 5.85063749 | -2.545759 | 2.58E-06 | 7.12E-06 |
| hsa-mir-937 | 17.3204903 | 3.06635593 | -2.4978827 | 8.17E-06 | 1.73E-05 |
| hsa-mir-589 | 309.383013 | 55.2842196 | -2.4844543 | 1.27E-06 | 5.94E-06 |
| hsa-mir-181a-2 | 5584.49963 | 999.299083 | -2.4824396 | 1.45E-06 | 5.94E-06 |
| hsa-mir-15b | 1203.91864 | 223.133235 | -2.4317606 | 1.29E-06 | 5.94E-06 |
| hsa-mir-744 | 222.52275 | 43.1125581 | -2.3677728 | 1.66E-06 | 5.94E-06 |
| hsa-mir-3074 | 37.1761388 | 7.59386 | -2.2914716 | 3.93E-06 | 9.64E-06 |
| hsa-mir-4746 | 16.008806 | 3.27809331 | -2.2879369 | 1.17E-05 | 2.37E-05 |
| hsa-mir-92a-1 | 49076.1275 | 10398.5451 | -2.2386397 | 1.61E-06 | 5.94E-06 |
| hsa-mir-887 | 10.5989935 | 2.26456138 | -2.2266237 | 1.76E-06 | 5.94E-06 |
| hsa-mir-3614 | 18.3800925 | 3.9581158 | -2.2152583 | 2.90E-06 | 7.72E-06 |
| hsa-mir-92a-2 | 44316.37 | 9627.87299 | -2.2025507 | 1.64E-06 | 5.94E-06 |
| hsa-mir-132 | 386.24935 | 84.3030374 | -2.195876 | 1.69E-06 | 5.94E-06 |
| hsa-mir-4326 | 32.1037324 | 7.21576489 | -2.1535168 | 2.62E-05 | 4.91E-05 |
| hsa-mir-140 | 2945.0095 | 665.041689 | -2.1467556 | 1.61E-06 | 5.94E-06 |
| hsa-mir-877 | 10.2487799 | 2.39275441 | -2.0987079 | 0.00022805 | 0.00034525 |
| hsa-mir-370 | 37.0442638 | 8.87990655 | -2.0606338 | 1.90E-06 | 5.97E-06 |
| hsa-mir-652 | 123.347751 | 29.8020624 | -2.0492473 | 1.54E-06 | 5.94E-06 |
| hsa-mir-200c | 49230.2425 | 12078.6611 | -2.0270843 | 1.33E-06 | 5.94E-06 |
| hsa-mir-1266 | 66.816655 | 16.3948914 | -2.0269614 | 5.69E-06 | 1.31E-05 |
| hsa-mir-664b | 10.703639 | 2.71740908 | -1.9777977 | 1.80E-05 | 3.55E-05 |
| hsa-mir-339 | 187.867264 | 48.3396297 | -1.9584354 | 7.96E-06 | 1.71E-05 |
| hsa-mir-3653 | 43.2276475 | 11.2681017 | -1.9397098 | 0.000127 | 0.00020108 |
| hsa-mir-891a | 6.21347913 | 1.63409028 | -1.9269136 | 0.00010595 | 0.00016916 |
| hsa-mir-342 | 292.679725 | 78.8718417 | -1.8917406 | 5.93E-06 | 1.35E-05 |
| hsa-mir-219a-1 | 15.0325624 | 4.14790319 | -1.8576368 | 4.04E-06 | 9.77E-06 |
| hsa-mir-323a | 14.6568335 | 4.15289236 | -1.8193851 | 6.10E-06 | 1.38E-05 |
| hsa-mir-501 | 218.74929 | 62.1411982 | -1.8156564 | 9.94E-06 | 2.04E-05 |
| hsa-mir-431 | 37.86629 | 10.8733097 | -1.8001229 | 3.21E-06 | 8.14E-06 |
| hsa-mir-3130-2 | 12.764821 | 3.81634963 | -1.7419081 | 6.27E-05 | 0.00010547 |
| hsa-mir-940 | 12.0214764 | 3.6103238 | -1.7354139 | 0.01459353 | 0.01806366 |
| hsa-mir-320a | 1465.03389 | 444.810483 | -1.7196713 | 5.93E-06 | 1.35E-05 |
| hsa-mir-361 | 955.815463 | 292.700888 | -1.707305 | 2.01E-06 | 6.12E-06 |
| hsa-mir-331 | 90.01388 | 27.823375 | -1.6938501 | 1.08E-05 | 2.20E-05 |
| hsa-mir-505 | 184.041611 | 57.0269401 | -1.6903165 | 6.55E-06 | 1.46E-05 |
| hsa-mir-1271 | 8.15699238 | 2.67696596 | -1.6074385 | 4.88E-05 | 8.46E-05 |
| hsa-mir-3651 | 9.34480425 | 3.0929664 | -1.5951733 | 0.00074391 | 0.00102422 |
| hsa-let-7a-2 | 18477.1563 | 6363.51369 | -1.5378472 | 1.84E-06 | 5.94E-06 |
| hsa-let-7a-3 | 18517.5863 | 6394.60988 | -1.5339678 | 1.76E-06 | 5.94E-06 |
| hsa-let-7a-1 | 18441.8775 | 6377.89414 | -1.5318335 | 1.74E-06 | 5.94E-06 |
| hsa-mir-1301 | 18.786685 | 6.55654375 | -1.5187031 | 8.07E-06 | 1.71E-05 |
| hsa-mir-3130-1 | 11.3086144 | 3.9864293 | -1.5042532 | 8.79E-05 | 0.00014401 |
| hsa-mir-4449 | 6.3512605 | 2.2433006 | -1.50142 | 0.00028775 | 0.00042217 |
| hsa-mir-127 | 2375.724 | 853.746382 | -1.4764878 | 2.90E-06 | 7.72E-06 |
| hsa-mir-3652 | 6.69405475 | 2.43639012 | -1.4581352 | 0.00042628 | 0.00060897 |
| hsa-mir-181b-2 | 258.881284 | 94.6705351 | -1.4513033 | 9.71E-05 | 0.00015704 |
| hsa-mir-151b | 2.75729075 | 1.03946957 | -1.4074039 | 0.02772683 | 0.03334239 |
| hsa-mir-28 | 13253.4238 | 5031.9728 | -1.3971691 | 1.76E-06 | 5.94E-06 |
| hsa-mir-664a | 42.1848888 | 16.5341887 | -1.351274 | 0.00014156 | 0.00021957 |
| hsa-mir-939 | 4.10886 | 1.61224041 | -1.3496713 | 0.00012973 | 0.0002043 |
| hsa-mir-3934 | 7.11626675 | 2.79879304 | -1.3463158 | 0.00036048 | 0.00052084 |
| hsa-mir-3200 | 11.2931299 | 4.54919769 | -1.3117613 | 0.00087065 | 0.0011901 |
| hsa-mir-363 | 9.89415263 | 4.10202104 | -1.2702413 | 0.00027964 | 0.00041187 |
| hsa-mir-1248 | 9.95900825 | 4.1376054 | -1.267206 | 0.00050508 | 0.00071627 |
| hsa-mir-181b-1 | 256.035473 | 107.612899 | -1.2504927 | 0.00022222 | 0.00033778 |
| hsa-mir-365a | 66.4037525 | 27.943652 | -1.2487442 | 5.90E-05 | 0.00010055 |
| hsa-mir-365b | 64.6326813 | 27.8567143 | -1.2142387 | 0.0001382 | 0.00021523 |
| hsa-mir-330 | 60.9101825 | 26.2821154 | -1.212602 | 3.23E-05 | 5.93E-05 |
| hsa-mir-543 | 3.23279825 | 1.39916978 | -1.2082124 | 0.00431285 | 0.00551813 |
| hsa-mir-30d | 12390.0225 | 5411.22155 | -1.1951526 | 4.73E-06 | 1.10E-05 |
| hsa-let-7e | 1273.82344 | 562.801242 | -1.1784679 | 0.00012547 | 0.00019949 |
| hsa-mir-491 | 6.54183538 | 2.92417646 | -1.1616651 | 0.01471871 | 0.01815945 |
| hsa-mir-1287 | 39.7268725 | 17.8959372 | -1.1504831 | 8.42E-06 | 1.76E-05 |
| hsa-mir-935 | 17.4562873 | 7.96868474 | -1.1313332 | 0.00123138 | 0.00166521 |
| hsa-mir-191 | 1660.07258 | 763.353549 | -1.120823 | 3.54E-05 | 6.40E-05 |
| hsa-mir-214 | 50.4010713 | 24.5924853 | -1.0352369 | 0.0002356 | 0.00035526 |
| hsa-mir-550a-3 | 3.35751388 | 1.65530772 | -1.0202939 | 0.00947189 | 0.01183986 |
| hsa-mir-497 | 25.6500863 | 12.6675545 | -1.0178256 | 0.00058964 | 0.00082376 |
| hsa-mir-625 | 1039.44241 | 517.979979 | -1.0048416 | 0.00058319 | 0.00081776 |
| hsa-mir-493 | 6.74727475 | 14.2408872 | 1.07766222 | 0.00417201 | 0.00535596 |
| hsa-mir-186 | 140.363754 | 299.803507 | 1.09484682 | 0.00023836 | 0.00035801 |
| hsa-mir-185 | 26.3530325 | 57.4553996 | 1.1244735 | 0.00013011 | 0.0002043 |
| hsa-let-7g | 285.954875 | 645.77944 | 1.17525401 | 3.36E-05 | 6.11E-05 |
| hsa-mir-130a | 17.0817043 | 39.6589308 | 1.21519386 | 0.00127286 | 0.0017152 |
| hsa-mir-218-1 | 3.77206813 | 9.12859038 | 1.27503637 | 0.00765502 | 0.00960035 |
| hsa-mir-576 | 5.15948 | 12.8458334 | 1.31600292 | 0.00405587 | 0.00522451 |
| hsa-mir-26a-1 | 324.791588 | 860.183995 | 1.40513102 | 8.07E-06 | 1.71E-05 |
| hsa-mir-26a-2 | 322.973738 | 860.967768 | 1.41454237 | 7.32E-06 | 1.63E-05 |
| hsa-mir-22 | 17491.4113 | 47033.7911 | 1.42705093 | 4.53E-06 | 1.07E-05 |
| hsa-mir-412 | 2.21711113 | 6.11093671 | 1.46271246 | 0.01676606 | 0.02048586 |
| hsa-mir-151a | 924.134025 | 2653.32341 | 1.52162653 | 5.45E-06 | 1.26E-05 |
| hsa-mir-146a | 87.3420788 | 251.400486 | 1.52523867 | 0.0018777 | 0.00247752 |
| hsa-mir-194-1 | 3087.55954 | 8973.9952 | 1.53928346 | 0.00010453 | 0.0001676 |
| hsa-mir-31 | 7.37320963 | 21.9295667 | 1.57251263 | 0.00172172 | 0.0022876 |
| hsa-mir-34a | 38.87483 | 121.030442 | 1.6384617 | 7.04E-05 | 0.00011675 |
| hsa-mir-30e | 2993.48838 | 9523.19538 | 1.66961806 | 1.84E-06 | 5.94E-06 |
| hsa-mir-181d | 6.04457475 | 19.911269 | 1.71987242 | 0.00093848 | 0.00127365 |
| hsa-mir-584 | 103.90339 | 347.47924 | 1.74168406 | 0.00060273 | 0.0008359 |
| hsa-mir-196a-1 | 44.6348713 | 150.64497 | 1.75490933 | 0.00053394 | 0.00075427 |
| hsa-mir-10b | 11216.2388 | 38601.1509 | 1.7830549 | 8.07E-06 | 1.71E-05 |
| hsa-mir-24-1 | 296.888275 | 1031.86242 | 1.79725861 | 2.17E-06 | 6.39E-06 |
| hsa-mir-199a-1 | 347.2586 | 1248.55381 | 1.84617567 | 2.55E-05 | 4.82E-05 |
| hsa-mir-224 | 25.253052 | 92.599534 | 1.87454718 | 0.00024972 | 0.00037213 |
| hsa-mir-18a | 10.18331 | 38.002207 | 1.89987663 | 0.00057681 | 0.00081181 |
| hsa-mir-26b | 179.560206 | 678.253105 | 1.91735609 | 3.45E-06 | 8.63E-06 |
| hsa-mir-24-2 | 273.916063 | 1039.06583 | 1.92348129 | 1.57E-06 | 5.94E-06 |
| hsa-mir-147b | 1.60741113 | 6.16623503 | 1.93965091 | 0.01007442 | 0.01255174 |
| hsa-mir-7974 | 0.31342275 | 1.21588187 | 1.95582126 | 0.02873901 | 0.03445055 |
| hsa-mir-27a | 452.34255 | 1771.42584 | 1.96942346 | 3.82E-06 | 9.49E-06 |
| hsa-mir-196b | 847.829713 | 3844.81417 | 2.18106744 | 0.00290174 | 0.00377624 |
| hsa-mir-199a-2 | 493.6188 | 2243.43549 | 2.18424046 | 7.85E-06 | 1.71E-05 |
| hsa-mir-338 | 116.817761 | 532.079728 | 2.1873828 | 0.00021204 | 0.0003236 |
| hsa-mir-216a | 0.25994363 | 1.25882673 | 2.27580904 | 0.00278136 | 0.00363202 |
| hsa-mir-340 | 3.849157 | 18.8177943 | 2.28948312 | 2.70E-06 | 7.38E-06 |
| hsa-mir-3677 | 2.056434 | 10.3625871 | 2.33316755 | 9.36E-05 | 0.00015265 |
| hsa-mir-29c | 209.711188 | 1060.83004 | 2.3387178 | 6.55E-06 | 1.46E-05 |
| hsa-mir-337 | 4.74684475 | 26.8525868 | 2.5000203 | 3.21E-06 | 8.14E-06 |
| hsa-mir-223 | 71.5807075 | 406.232902 | 2.50466439 | 4.24E-05 | 7.42E-05 |
| hsa-mir-29b-2 | 82.3432488 | 471.841323 | 2.5185795 | 2.95E-06 | 7.77E-06 |
| hsa-mir-183 | 1547.91438 | 8911.56533 | 2.5253532 | 3.12E-06 | 8.02E-06 |
| hsa-mir-95 | 2.97125113 | 17.5249379 | 2.56026688 | 5.94E-05 | 0.00010073 |
| hsa-mir-196a-2 | 25.845825 | 153.719779 | 2.57229965 | 3.82E-05 | 6.85E-05 |
| hsa-mir-451a | 55.7159675 | 334.22383 | 2.58465185 | 9.71E-05 | 0.00015704 |
| hsa-mir-190a | 1.04634763 | 6.35197316 | 2.60184258 | 2.33E-05 | 4.49E-05 |
| hsa-mir-143 | 24061.3725 | 149204.773 | 2.63250284 | 2.04E-05 | 3.97E-05 |
| hsa-mir-218-2 | 1.36400225 | 8.65672396 | 2.66597513 | 3.07E-05 | 5.65E-05 |
| hsa-mir-192 | 12371.8019 | 78966.1836 | 2.67417933 | 1.48E-05 | 2.95E-05 |
| hsa-mir-33a | 11.1770875 | 71.5974807 | 2.67936452 | 9.95E-05 | 0.00016026 |
| hsa-mir-7-1 | 9.08919438 | 61.5408271 | 2.7593195 | 1.99E-06 | 6.12E-06 |
| hsa-mir-10a | 11170.4646 | 76626.2686 | 2.77814986 | 2.14E-06 | 6.34E-06 |
| hsa-mir-627 | 0.40426488 | 2.81923063 | 2.80192874 | 0.00019968 | 0.00030597 |
| hsa-mir-30b | 51.9102913 | 369.917527 | 2.83311117 | 1.74E-06 | 5.94E-06 |
| hsa-mir-199b | 425.0089 | 3083.94822 | 2.85921359 | 2.14E-06 | 6.34E-06 |
| hsa-mir-17 | 208.151173 | 1527.91518 | 2.87586087 | 2.11E-06 | 6.34E-06 |
| hsa-mir-188 | 1.18143888 | 8.91283712 | 2.91533975 | 2.82E-05 | 5.24E-05 |
| hsa-mir-598 | 1.83794675 | 14.2305298 | 2.9528225 | 2.31E-05 | 4.48E-05 |
| hsa-mir-33b | 1.0275795 | 8.39735929 | 3.0306857 | 0.00026007 | 0.00038454 |
| hsa-mir-496 | 0.31251188 | 2.65598243 | 3.08726269 | 3.72E-05 | 6.70E-05 |
| hsa-mir-4662a | 1.37452475 | 11.9711724 | 3.12255966 | 6.86E-05 | 0.00011436 |
| hsa-mir-32 | 4.2470485 | 38.9353191 | 3.19654696 | 1.66E-06 | 5.94E-06 |
| hsa-mir-15a | 14.4507043 | 133.653344 | 3.20928422 | 1.27E-06 | 5.94E-06 |
| hsa-mir-369 | 1.43990363 | 13.3696515 | 3.2149177 | 2.90E-06 | 7.72E-06 |
| hsa-mir-152 | 20.7534638 | 200.302874 | 3.27075907 | 1.27E-06 | 5.94E-06 |
| hsa-mir-495 | 0.698769 | 6.77990258 | 3.27837703 | 2.33E-06 | 6.60E-06 |
| hsa-let-7f-1 | 387.458775 | 3956.35695 | 3.35205787 | 2.20E-06 | 6.43E-06 |
| hsa-mir-429 | 43.5448475 | 447.849323 | 3.3624395 | 2.27E-06 | 6.57E-06 |
| hsa-let-7f-2 | 382.45955 | 4008.54997 | 3.38970138 | 1.82E-06 | 5.94E-06 |
| hsa-mir-5000 | 0.183451 | 1.92966636 | 3.39488475 | 5.49E-05 | 9.43E-05 |
| hsa-mir-148a | 6728.31525 | 71855.9806 | 3.41679103 | 1.52E-06 | 5.94E-06 |
| hsa-mir-217 | 3.37886688 | 38.82532 | 3.5223864 | 9.40E-06 | 1.94E-05 |
| hsa-mir-126 | 199.756009 | 2338.49182 | 3.54926758 | 1.27E-06 | 5.94E-06 |
| hsa-mir-3613 | 1.35782188 | 16.5228155 | 3.6050934 | 4.16E-06 | 9.89E-06 |
| hsa-mir-7-3 | 0.365909 | 4.67032535 | 3.67396625 | 3.92E-05 | 6.92E-05 |
| hsa-mir-421 | 0.40403713 | 5.27211784 | 3.70582285 | 7.63E-06 | 1.69E-05 |
| hsa-mir-660 | 4.0713805 | 53.9940261 | 3.72920983 | 1.35E-06 | 5.94E-06 |
| hsa-mir-494 | 0.13008575 | 1.73610258 | 3.73831735 | 6.05E-05 | 0.00010223 |
| hsa-mir-106a | 3.90211063 | 53.2391775 | 3.7701617 | 4.13E-05 | 7.27E-05 |
| hsa-mir-374b | 5.59894625 | 77.2023803 | 3.78541809 | 1.35E-06 | 5.94E-06 |
| hsa-mir-376b | 0.14064463 | 1.96581208 | 3.80499909 | 6.83E-05 | 0.00011428 |
| hsa-mir-651 | 0.25994363 | 3.70977456 | 3.83506084 | 1.85E-05 | 3.63E-05 |
| hsa-mir-655 | 0.13008575 | 1.95351468 | 3.90853726 | 5.02E-05 | 8.68E-05 |
| hsa-mir-16-1 | 25.1991075 | 378.504353 | 3.90886535 | 1.27E-06 | 5.94E-06 |
| hsa-mir-98 | 3.5655245 | 53.9043032 | 3.91821423 | 1.27E-06 | 5.94E-06 |
| hsa-mir-508 | 2.53502188 | 39.1103103 | 3.94747888 | 0.01623527 | 0.01996571 |
| hsa-mir-16-2 | 24.7330231 | 382.937356 | 3.95259791 | 1.27E-06 | 5.94E-06 |
| hsa-mir-379 | 51.4914963 | 803.190823 | 3.96333669 | 1.27E-06 | 5.94E-06 |
| hsa-mir-2355 | 2.33260075 | 37.6701753 | 4.01341145 | 1.27E-06 | 5.94E-06 |
| hsa-mir-4677 | 0.448628 | 7.27651133 | 4.01965536 | 1.69E-06 | 5.94E-06 |
| hsa-mir-301b | 0.18868438 | 3.21739878 | 4.0918479 | 2.53E-05 | 4.81E-05 |
| hsa-mir-1-2 | 1.34222713 | 23.5644106 | 4.13390888 | 1.24E-05 | 2.50E-05 |
| hsa-mir-215 | 57.094035 | 1044.37301 | 4.19315324 | 9.15E-06 | 1.90E-05 |
| hsa-mir-203a | 1233.41549 | 22696.1138 | 4.20171452 | 2.01E-06 | 6.12E-06 |
| hsa-mir-136 | 1.85687813 | 37.5490604 | 4.33782578 | 1.59E-06 | 5.94E-06 |
| hsa-mir-411 | 0.38991538 | 8.45465058 | 4.43851218 | 3.99E-06 | 9.71E-06 |
| hsa-mir-452 | 5.44213938 | 124.16325 | 4.51192051 | 1.27E-06 | 5.94E-06 |
| hsa-mir-335 | 10.063647 | 235.381386 | 4.54777511 | 1.27E-06 | 5.94E-06 |
| hsa-mir-182 | 724.53565 | 16968.3276 | 4.54964389 | 1.31E-06 | 5.94E-06 |
| hsa-mir-141 | 75.096905 | 1947.64045 | 4.69683011 | 1.27E-06 | 5.94E-06 |
| hsa-mir-552 | 8.07778113 | 210.457884 | 4.70342869 | 1.36E-05 | 2.74E-05 |
| hsa-mir-20a | 21.2453786 | 593.389462 | 4.80375835 | 1.27E-06 | 5.94E-06 |
| hsa-mir-96 | 0.75126275 | 21.072596 | 4.80990667 | 1.76E-06 | 5.94E-06 |
| hsa-mir-301a | 0.57859975 | 16.2901505 | 4.81529042 | 3.08E-06 | 7.95E-06 |
| hsa-mir-29b-1 | 15.886905 | 470.76794 | 4.88910608 | 1.27E-06 | 5.94E-06 |
| hsa-mir-1-1 | 0.70491325 | 22.2984152 | 4.98335165 | 3.93E-06 | 9.64E-06 |
| hsa-mir-101-2 | 101.614503 | 3297.22265 | 5.02007309 | 1.27E-06 | 5.94E-06 |
| hsa-mir-101-1 | 99.3697988 | 3275.75607 | 5.04287668 | 1.27E-06 | 5.94E-06 |
| hsa-mir-424 | 4.70824163 | 155.372908 | 5.0444028 | 1.27E-06 | 5.94E-06 |
| hsa-mir-582 | 12.8284635 | 426.665906 | 5.05568654 | 1.29E-06 | 5.94E-06 |
| hsa-mir-628 | 0.640917 | 23.4360766 | 5.19244972 | 1.66E-06 | 5.94E-06 |
| hsa-mir-708 | 1.54265975 | 61.4228572 | 5.31528382 | 1.31E-06 | 5.94E-06 |
| hsa-mir-144 | 2.04453025 | 82.0489754 | 5.32664401 | 1.39E-06 | 5.94E-06 |
| hsa-mir-542 | 4.02702913 | 164.609211 | 5.35318535 | 1.27E-06 | 5.94E-06 |
| hsa-mir-889 | 0.37736875 | 16.1915287 | 5.42312044 | 1.31E-06 | 5.94E-06 |
| hsa-mir-376c | 0.14064463 | 6.23483324 | 5.47022465 | 2.45E-06 | 6.86E-06 |
| hsa-mir-153-2 | 0.95069488 | 45.1508707 | 5.56962762 | 1.74E-06 | 5.94E-06 |
| hsa-mir-203b | 4.13973575 | 218.343514 | 5.72091719 | 1.71E-06 | 5.94E-06 |
| hsa-mir-21 | 3824.411 | 208937.031 | 5.77168683 | 1.27E-06 | 5.94E-06 |
| hsa-mir-454 | 0.1440935 | 8.96151742 | 5.95866588 | 1.38E-06 | 5.94E-06 |
| hsa-mir-19b-1 | 1.99172138 | 146.773684 | 6.20343367 | 1.27E-06 | 5.94E-06 |
| hsa-mir-142 | 31.2540675 | 3018.43912 | 6.59361493 | 1.27E-06 | 5.94E-06 |
| hsa-mir-19b-2 | 1.09078875 | 129.395254 | 6.89026917 | 1.27E-06 | 5.94E-06 |
| hsa-mir-374a | 5.62217 | 1023.34907 | 7.50795554 | 1.27E-06 | 5.94E-06 |
| hsa-mir-577 | 0.68877538 | 126.076361 | 7.51604852 | 1.61E-06 | 5.94E-06 |
| hsa-mir-590 | 0.13008575 | 29.5351582 | 7.8268266 | 1.45E-06 | 5.94E-06 |
| hsa-mir-135b | 0.1440935 | 109.600642 | 9.57103527 | 1.29E-06 | 5.94E-06 |
